# Immune System and Inflammation

**Published:** 2008-10

**396**

**Evaluation of patterns of use of drugs for management of rheumatoid arthritis in patients attending rheumatology opd in B.Y.L. Nair charitable hospital**

Jadhav A, Bhide S^1^

**^1^Topiwala National Medical College and B.Y.L. Nair Hospital, Mumbai, Department of Pharmacology, Topiwala National Medical College, Mumbai, India.**

**Introduction:**

Rheumatoid arthritis is an autoimmune chronic inflammatory disease characterized by pain, swelling, stiffness and destruction of joints due to synovitis with resultant disability. It affects 0.5-1% of the industrialized population. Very few studies are being conducted on drug utilization pattern in rheumatoid arthritis.

**Methods:**

Patients with Rheumatoid arthritis attending Rheumatology OPD of B Y.L. Nair Hospital were interviewed over a period of 4 months. Information was obtained from these interviews and patients OPD papers to obtain detailed drug history, any ADR and severity of disease.

**Results:**

Out of 69 patients interviewed, majority were females (55). The average group was 43 years. 31 patients were taking treatment since 2 years and less. Most common drug among DMARD’S is Methotraxate, followed by Chloroquine; while the most common drug among NSAID’S is Indomethacin. No Biological drugs were prescribed. ADR was reported in 2 patients.

**Conclusions:**

Methotraxate was the most commonly prescribed DMARD which is in accordance with Indian guidelines.

**397**

**Study on bioactive compounds and their antimicrobial activity in *Withania somnifera***

Goswami M^1^, Sharma RA^2^, Kumari B^2^

**^1^University of Rajasthan, Jaipur, India; ^2^University of Rajasthan, Jaipur, India.**

Ashwagandha is a very significant herbal drug in Ayurveda or Indian System of medication. This meticulous herb was used to treat a variety of infectious diseases as well as tremors and inflammation especially arthritis. *Ashwagandha* has comparable properties as Chinese ginseng. The active components in *Ashwagandha* which provides it with the properties it possesses and it is the only the secondary metabolite which is having the medicinal character. Therefore, *Ashwagandha* is known to kindle the immune system, stop inflammation, increase memory, and helps maintain general healthy and wellness. In this present study we had studied the property of Ashwagandha, identified the secondary metabolite (alkaloids, flavinoids) present in plant of Ashwagandha by biochemical process Studied the biochemical profile, we did the sequential extraction and isolation of various metabolites from the crude extracts Identification, Quantification and its antimicrobial activity of bioactive compounds. By knowing the secondary metabolite (Alkaloid, flavonoids,) of the plant we can use this in the industrial basis to develop new drugs. Our aim is develop our rural to Industrial knowledge.

**398**

**Protective effect of a combination of *Tinospora cordifolia* and *Phyllanthus emblica* with and without *Ocimum sanctum* against infection in normal and immunosuppressed animals: An experimental study**

Shankar S, Kamat SK, Rege NN

**Seth GS Medical College and KEM Hospital, Parel, Mumbai, India.**

**Introduction:**

The study was undertaken to evaluate whether a combination of *Tc* and *Pe* (1:3) with or without *Os* coating offers protection against infection in normal and immunosuppressed animals.

**Methods:**

Animal Ethics Committee approval was obtained. The study was conducted in 2 models of infection. In each model, 64 mice were divided into 8 groups (n=8/gp) namely; Gp1:vehicle, Gp2:*Tc*(100mg/kg/d), Gp3:*Tc+Pe*(400mg/kg/d), Gp4:Tc+Pe+Os(400mg/kg/d), Gp5:Antibiotic(given at ED50), Gp6:Antibiotic+*Tc*, Gp7:Antibiotic+*Tc+Pe*, Gp8:Antibiotic+ *Tc+Pe+Os*. Mice were pre-treated with the vehicle/test drugs for 15 days. On D16, in model 1, *E.coli* (10^8^ CFU) was injected i.p. Gentamicin (1.2mg/Kg; s.c.) was administered 1 hr later. In the model of immunosupression, on D16 cyclophosphamide was injected (200 mg/Kg; i.p). Test drugs/vehicle were continued till injection of *S.aureus* (10^9^ CFU; i.p.) on D19. Sparfloxacin (6.3mg/ kg) was administered orally 1 hr and 4 hrs later. Mice were observed for mortality at the end of 24 and 48 hrs.

**Results:**

In both the models the plant formulations *per se* did not significantly improve survival as compared to vehicle. However, when combined with gentamicin, both *Tc+Pe* and *Tc+Pe+Os* showed a significant improvement in survival against Ecoli infection at 48 hrs as compared to gentamicin alone (87.5% vs 37.5%; *P*<0.05). Similarly in model 2, addition of sparfloxacin to *Tc+Pe+Os* improved survival of immunosuppressed mice against staphylococcal infection at the end of 48 hrs (87.5% *vs* 37.5%; *P*<0.05)as compared with sparfloxacin alone.

**Conclusions:**

The selected plant formulations appear to be useful as adjuvants in the treatment of infections.

**399**

**Targeting inflammation by PPAR-gamma agonist in LPS induced pulmonary dysfunction**

Sharma R, Kaundal R K, Kumar A, Sharma SS

**National Institute of Pharmaceutical Education and Research (NIPER), Sector 67, SAS Nagar, Mohali-160062, India.**

**Introduction:**

Pulmonary dysfunction and pulmonary neutrophilic inflammation are the major characteristics of inflammatory conditions of lungs like chronic obstructive pulmonary disease (COPD). In the present study, we have evaluated the effects of pioglitazone, a peroxisome proliferator-activated receptors gamma (PPARγ) agonist, in LPS-induced pulmonary dysfunction, inflammatory changes and oxidative stress in guinea pigs.

**Materials and Methods:**

Guinea pigs were exposed to nebulised lipopolysaccharide (30 *μ*g ml^-1^, 0.45 ml min^-1^) for one hour in a Perspex exposure chamber and lung function parameters were measured after LPS or saline exposure. After 24 hour, bronchoalveolar lavage was done and inflammatory cell influx as well as TNF-alpha levels was estimated in the pooled lavage fluid. Additionally, to confirm neutrophil migration in lung tissue, we measured tissue myeloperoxidase activity (MPO). Malonaldialdehyde (MDA) estimation was done as an index of lipid peroxidation. Lung Histology was performed and sections observed for inflammatory changes.

**Results:**

Results from the present study demonstrated that the LPS-induced pulmonary dysfunction was temporally associated with neutrophil infiltration as evident from heavy neutrophilia, increased TNF-alpha levels, elevated MPO and histology of the lung tissue and increased oxidative stress. The results also reveal that pioglitazone (3, 10 and 30 mg kg-1, p.o.) is effective in abrogating the pulmonary dysfunction by attenuating pulmonary dysfunction, neutrophilia, TNF-alpha release and oxidative stress in LPS induced model of acute lung inflammation.

**Conclusions:**

Present study embodied evidences of PPARγ agonists effectiveness in the therapy for inflammatory disease of the lungs.

**400**

**Effect of a topical curcumin preparation on burn wound healing in rats**

Safalya DP^1^, Rai RK^2^, Reddy HK^2^

**^1^KMC International Centre, Manipal, India; ^2^Kasturba Medical College, Manipal, India.**

Wound healing is a complex multifactorial process that results in the contraction and closure of the wound and restoration of a functional barrier. Repair of injured tissues occurs as a sequence of events, which include inflammation, proliferation, and migration of different cell types. Curcumin, a naturally occurring *o*-methoxyphenol derivative, has been shown to possess several biological properties including antioxidant (free radical scavenging activity), induction of detoxification enzymes and provides protection against degenerative diseases. Topical applications of compounds with free radical scavenging properties in patients have shown to improve significantly wound healing and protect tissues from oxidative damage. The aim of this study the assess the effect of a topical curcumin preparation on healing of burn wounds in rats. Materials and Methods:The rats are randomly divided into four groups, comprising of six rats in each group. Partial thickness burn wounds are created by pouring hot molten wax at 80°C. Group I acts a control, Group 2 recieves the standard silver sulphadiazine cream, Group 3 gets 20% curcumin cream, and Group 4 recieves the combination of the standard and the active drug. Parameters observed are epithelialization period and wound contraction. Results: Results are awaited as the study is ongoing.

**401**

**Study of efficacy of oral dapsone in patients of lichen planus as compared to topical steroids**

Sharma A, Chugh Y, Kastury N, Tiwari AR, Kapoor AK, Singh KG

**MLN. Medical College, Allahabad, India.**

**Objective:**

To study the efficacy of oral dapsone as compared to topical steroids in the patients attending the out patient department of Skin and VD of S.R.N Hospital attached to MLN Medical College, Allahabad.

**Materials and Methods:**

The study was conducted from August 2007 to June 2008. Clinically diagnosed patients of Lichen Planus were randomly selected and divided in two groups; R1 received *dapsone* (100 mg BD) + anti-histaminics whereas R2 treated with topical steroid + anti-histaminics. Clinical interpretation was made by observing reduction of itching, regression of the size and shape of papules and appearance of new lesions.

**Results:**

Out of 200 patients, 60% belonged to age group 30-60 years and only 4% cases were less than 10 years. Male to female ratio was 1.08:1. Among these selected patients, 93 were given R1 and 107 received R2. They were followed up for three months. After 3 months of therapy, good response was observed in 58% patients receiving R1 where as in 40% of R2. No age/sex wise difference was seen in clinical improvement.

**Discussion and conclusion:**

Dapsone has a therapeutic effect in several dermatoses and in Lichen Planus. The use of dapsone has been recommended by several dermatologists. It is used in dermatology for its anti-inflammatory properties. It may be due to inhibition of myeloperoxidase hydrogen peroxide cytotoxic system. Our study reveals that Dapsone is definitely superior to local corticosteroids in treating Lichen Planus cases.

**402**

**Adaptogenic activity of ethanolic extract of leaves of *Rhododendron arboreum* in mice and rats**

Swamidasan R, Prakash T, Divakar Gol, Kamalesh DR, Dixit P, Chandrasekar SB

**Acharya and B.M. Reddy College of Pharmacy. Bangalore-560-090, India.**

**Objective:**

Stress is a non-specific response of the body known to alter the physiological homeostasis of the organism resulting in various neuronal, endocrinal and visceral functions. Derailment of the immune system contributes for the alteration in the homeostasis and resulting in the stress related disturbances. Adaptogen is an agent that allows body to counter adverse physical, chemical or biological stress by raising non-specific resistance towards stress, thus allowing the organism to adapt stressful circumstances. So the present study was undertaken to evaluate the adaptogenic activity (anti-stress) of the ethanolic extract of *R. arboreum*.

**Materials and Methods:**

The study was conducted on mice and rats. Anoxia stress tolerance, Swimming endurance, Immobilization stress models were used for the evaluation of adaptogenic activity. Group I served as control (vehicle alone). Group II and III was pretreated with extract at doses 250 and 500 mg/kg p.o. Group IV was treated with *Withania somnifera* (100mg/kg, p.o) as standard.

**Results:**

Concomitant treatment with ethanolic extract at doses 250 and 500 mg/kg, showed marked increase in anoxia stress tolerance and swimming endurance time as compared to control group. Similarly, pre-treatment with extract showed marked decrease in blood glucose, cholesterol, triglycerides level as compared to stress control group in Immobilization stress. Weights of liver and adrenal glands are markedly decreased, but no weight changes in spleen and testes were observed.

**Conclusions:**

The present investigation indicates that the extract of *R. arboreum* possess significant adaptogenic property as by mitigating the effect of acute and chronic stress induced biochemical and physiological perturbation.

**403**

**Comparative analysis of the levels of proinflammatory cytokines, matrix metalloproteinases and their inhibitors in the synovial fluid samples of rheumatoid and osteoarthritis patients**

Sharma G^1^, Upadhyay A^1^, Carroll GJ^2^, Jazayeri JA^1^

**^1^Monash University, Melbourne, Australia; ^2^Fremantle Hospital, Perth, Australia.**

Cytokines play a significant role in regulating or modifying the level of matrix metalloproteinases (MMPs) in the pathogenesis of arthritis. Cytokine levels in the synovial fluid samples of Rheumatoid Arthritis (RA) and Osteoarthritis (OA) patients have been regarded as biomarker of the disease severity, which could be used to scrutinize the process of inflammation and cartilage damage in the diseased joints. The aims of this study were to do, comparative analysis of the levels of different cytokines such as LIF, TNF-alpha, IL-1, IL-6 and OSM, in the synovial fluid samples aspirated from 10 Rheumatoid arthritis (RA), 25 Osteoarthritis (OA) and 7 Mixed arthropathies (MA) patients. The concentrations of these cytokines were determined by enzyme-linked immunosorbent assay (ELISA). In addition, the quantative expression profiling of MMP-3, tissue inhibitor of metalloproteinases 1 (TIMP-1) and MMP1/TIMP1 complex, which are mainly implicated in the pathogenesis of arthritis, was also carried out through ELISA. The results suggest that the level of MMPs and cytokines are significantly higher in RA than OA patients. Interestingly, elevated levels of cytokines such as IL-1, OSM and LIF were found in RA patients, whereas the concentrations of these cytokines were too low in OA and MA patients. The concentrations of cytokines quantified in MA patients were lower than RA but higher than OA patients. Furthermore, we found a significant correlation pattern between the levels of TIMP-1 and OSM. In conclusion, our study shows that, RA patients have significantly increased levels of cytokines and MMPs than MA and OA patients.

**404**

**Evaluation of hydroalcoholic extract of *Vitex negundo* L as an adjuvant therapy in Freund’s adjuvant induced arthritis**

Kagathara VG, Sannapuri VD, Vyawahare NS, Harle UN, Joshi VS

**AISSMS College of Pharmacy, Kennedy road, Pune, India.**

In this study, the anti-arthritic effect of hydro alcoholic extract of *Vitex negundo* L (VN) on Complete Freund’s adjuvant (CFA) induced arthritis has been studied in wistar albino rats. The hydroalcoholic extracts of *Vitex negundo* L. significantly reduced paw volume, inhibited body weight loss as compared to vehicle treated control rats. Moreover significant improvement in histological and radiographic parameters like cellular infiltration, pannus formation, joint space and soft tissue swelling were also observed. Similar results were also noted when it was combined with normal dose as well as reduced dose of synthetic agents like Indomethacin and Methotrexate. These results indicated that hydroalcoholic extract of *Vitex negundo* L shows anti-arthritic activity and constitutes to a safe, economical and potential adjuvant to primary therapeutic agents Indomethacin and Methotrexate for CFA.

**405**

**Anti-inflammatory screening of leaf extracts of *Syzygium alternifolium***

Gopi Chand, Sai Krishna, Gopi Sudheer, Raghavendra, Pradeep, Ranganayakulu D

**Sri Padmavathi School of Pharmacy, Tiruchanoor, Tirupathi, India.**

**Objective:**

To extract and to evaluate anti-inflammatory activity of leaf of *Syzygium alternifolium* in both acute and subacute models of inflammation using albino rats.

**Materials and Methods:**

The dried coarse leaf powder of *Syzygium alternifolium* was extracted successively with hexane, chloroform, alcohol using soxhlet and water by percolation. These extracts were used to perform acute toxicity stuies and also to evaluate anti inflammatory activity by acute model like carageenan induced paw oedema and subacute model like cotton pellet granuloma method.

**Results:**

All the leaf extracts were safe with no behavioural changes upto the dose of 500 mg/kg body weight. In the caraggenan induced paw oedema, a significant decrease in paw oedema was noted at the end of 3 hr that was maintained upto 6 hr with alcoholic extract of leaf of *Syzygium alternifolium*. The hexane and aqueous extracts also showed similar results comparable to that of standard drug Diclofenac sodium, but the chloroform extract did not produce significant reduction in the paw oedema. In the subacute model of inflammation caused by cotton pellet granuloma model the leaf extracts of *Syzygium alternifolium* were not effective except alcoholic extract that caused 52% of inhibition in the inflammation.

**Conclusions:**

The results indicate that identification of active principle from the leaves of *Syzygium alternifolium* may add a new, potential antiinflammatory drug in the present armamentarium of drugs to treat acute conditions.

**406**

**Experimental studies on the possible mechanism of action of UNIM-352, a polyherbal, anti-asthma preparation**

Guhathakurta S, Gulati K, Siddiqui MK^1^, Vijayan VK, Ray A

**Vallabhbhai Patel Chest Institute, University of Delhi, Delhi, and ^1^Central Council for Research in Unani Medicine (Dept. of AYUSH), New Delhi, India.**

**Objective:**

To study the possible mechanism of action of UNIM-352, a polyherbal, anti-asthma preparation, in rats.

**Materials and Methods:**

Wistar rats (150-200g, n=6 per group), immunized with Keyhole Limpet Hemocyanin (KLH) were used. The study protocol was approved by the Institutional Animal Ethical Committee (IAEC). These KLH immunized rats were treated orally for 15 days with UNIM-352 (200 and 400 mg/kg), and subsequently blood and bronchoalveolar lavage (BAL) samples were collected. These blood and BAL samples were biochemically analysed for TNF-alpha, superoxide dismutase (SOD) and catalase using commercially available ELISA kits. An additional group of rats were exposed to restraint stress (RS) and blood samples from this group were also assayed for the above mentioned biochemical markers. The data were analysed using the Mann-Whitney U test.

**Results:**

Analysis of the data showed that UNIM-352 dose dependently attenuated TNF-alpha levels in normal rats, the attenuations being more marked in the BAL fluid that in the blood. In RS exposed rats, TNFalpha levels were elevated as compared to those in normal (no RS) rats, and UNIM-352 was equally effective in lowering this proinflammatory cytokine level in both blood and BAL samples. UNIM-352 pretreatment differentially enhanced SOD and catalase levels, in blood and BAL – the changes being more marked in the latter group.

**Conclusions:**

The results are suggestive of the possible involvement of anti-inflammatory and anti-oxidant effects of UNIM-352, and may help to explain its efficacy in bronchial asthma.

**407**

**An epidemiological survey of arthritis in the population of north Gujarat**

Patel MM^1^, Patel NJ^2^, Patel RK^2^, Patel MM^3^, Patel RJ^2^, Shah KK^4^

**^1^B. S. Patel Pharmacy College, Mehsana, ^2^Shree S. K. Patel College of Pharmaceutical Education and Research, Kherva, ^3^Kalol Institute of Pharmacy and Research Centre, Kalol, ^4^Hemchandracharya North Gujarat University, Patan, India.**

**Purpose:**

A study was conducted for Arthritis, mainly focused on Osteoarthritis (OA) and Rheumatoid Arthritis (RA), to check the prevalence, awareness, severity, probable causes, drug utilization in population of North Gujarat.

**Methods:**

Detailed questionnaires were designed to collect data randomly from 260 patients of Palanpur and Mehsana districts.

**Results:**

Prevalence of OA was found to be 3.11% where as that of RA was 0.06%. Furthermore, the prevalence ratio (male: female) for OA was 1:1.19, where that of for RA was 1:4. The usage of Allopathic medicine for the treatment for OA and RA is highest as compared to other therapies. Family history for OA and RA patients was found 20% and 28.57% respectively. More numbers of parts affected in RA patients than that of OA suggesting severity and systemic effect of RA. Hence awareness of RA patients for pharmacological as well as non pharmacological treatment, knee replacement like surgery is higher than OA patients. Most of OA patients are just taking pain killer drugs (NSAIDs) irregularly without taking any therapy. Though RA is autoimmune disease patients did not prescribed newer DMARDs and monoclonal antibodies indicating cost factor and lack of awareness. Very few patients were undergone for the diagnostic tests like RA test but after diagnosis of RA, the patients were found more conscious for the regular checkup and treatment.

**Conclusions:**

It is reasonable to conclude that OA and RA both are most common inflammatory and chronic arthritis, which are responsible for high morbidity in the patients.

**408**

**Activity of *Parmelia perlata* (HUDS.) on inflammatory reaction: *In vitro – in vivo studies***

Sancheti JS, Fernandes BB, Jagtap AG

**Bombay College of Pharmacy, Mumbai, India.**

**Objective:**

A pharmacological investigation was carried out to evaluate the anti-inflammatory potential and the mechanism of action of *Parmelia perlata* (PP) in the experimental models. PP is a perennial lichen found on rocks or dead wood in temperate Himalayas which has been used for itching, ulcers, leprosy, headache in the folklore medicine.

**Methods:**

*In-vitro* analysis of ethanolic extract of PP (EPP) was done using lipoxygenase (LOX) reaction, nitric oxide (NO) and free radical scavenging. EPP was evaluated for *in-vivo* anti-inflammatory activity in carrageenan induced rat paw edema model (n=6) at 200, 400 and 600mg/kg (po). Carrageenan induced edema is a result of released mediators like serotonin, histamine, kinins, metabolites of arachidonic acids, COX, LOX, free radicals, including NO at different time points. Therefore, highest active dose in caraageenan induced edema (600 mg/kg) was further tested in arachidonic acid, bradykinin, histamine, and heat induced inflammatory models (n=6) to assess the possible mechanism of action.

**Results:**

EPP resulted in 67.44% lipoxygenase inhibition at 50 ug/ml, IC_50_ of 39.4 *μ*g/ml in NO scavenging assay and IC_50_ of 30.42 *μ*g/ml in free radical scavenging assay. EPP caused dose dependant inhibition in carrageenaninduced inflammation with maximum 61.86% inhibition at 600 mg/ kg (4 hr). Further EPP at 600 mg/kg showed inhibition of 63.64% (60 min), 52.63% (20 min), 68.18% (15 min) and 21.43% (30 min) in arachidonic acid, bradykinin, histamine and heat induced rat paw edema.

**Conclusions:**

Results of the present investigation signify the anti-inflammatory potential of EPP. Mechanistic evaluation revealed that the anti-inflammatory action of the extract may be due to inhibition of cycloxygenase, lipoxygenase nitric oxide, free radicals, histamine and bradykinin. Thus *Parmelia perlata* can be a good candidate for developing new herbal drug which can be used for various inflammatory conditions like rash, ulcers, rheumatoid arthritis, etc.

**409**

**Effect of tiotropium on carbachol-induced bronchoconstriction, salivation and bradycardia in anaesthetised guinea pigs**

Sinha S, Krishna NS, Raj Kumar S, Ray A

**Ranbaxy Research Laboratories, Gurgaon, India.**

The use of tiotropium is well established in the therapy of chronic obstructive pulmonary disease, but has side effects such as dry mouth and cardiac arrhythmias that result from lack of selectivity for the airways. The primary objective of this study was to determine the *in vivo* airway selectivity profile of tiotropium in anaesthetized guinea pigs by evaluating its effect on carbachol (CCh)-induced bronchospasm, salivary secretion and bradycardia. In anaesthetized guinea pigs, CCh-4*μ*g/kg elicited 181 and 163% increase in lung resistance (R_L_) from baseline at 2 and 24 h post intratracheal instillation of vehicle respectively. Tiotropium markedly reduced the CCh-induced increase in R_L_ with ID_90_ of 0.63 and 1.03*μ*g/kg at 2 and 24 h respectively. Tiotropium dose dependently inhibited CCh-3*μ*g/kg induced salivary secretion with 4.6 and 9.7 fold selectivity for the airways respectively at 2 and 24 h post-intstillation. Furthermore, it also showed a significant reversal of CCh-3*μ*g/kg induced bradycardia with 3.2 and 5.1 fold selectivity for the airways at 2 and 24 h post-intstillation respectively. These results indicate that tiotropium potently inhibits CCh-induced bronchoconstriction for greater than 24 h with marginal selectivity for airways vs. salivary gland and cardiac tissues in anesthetized guinea pigs.

**410**

**Evaluation of antiarthritic activity of majoon suranjan (a Unani compound formulation) in experimental model**

Singh Surender, Gupta YK, Nair Vinod, Jain Sweety

**Department of Pharmacology, All India Institute of Medical Sciences, New Delhi -29, India.**

**Background:**

Conventional agents used in the treatment of rheumatoid arthritis (RA) like NSAIDs, DMARDs and corticosteroids produce a plethora of side effects. Therefore, there has been an increase in the search of drugs in alternative medicine for treatment of RA. Majoon suranjan (MS) is a formulation mentioned in the Unani system of medicine for the treatment of RA. In the present study, efficacy of MS, was evaluated in an experimental model of arthritis in rats.

**Methods:**

Three groups (n=6) of Wistar albino rats (150-200g) were used in the study. Group I received distilled water (control), group II received MS (900mg/kg) and group III received standard drug aspirin (l00mg/kg). Initial joint size was measured using a micrometer screw gauge. After one day pretreatment, arthritis was induced by subplantar injection of 0.1 ml complete Freund’s adjuvant. The day of induction of arthritis was considered as day 1. Vehicle and drug treatment of the respective groups was continued for a period of 20 days. Joint size was measured on days 7, 14 and 21.

**Results and Conclusion:**

An initial increase in joint size was observed in all groups on day 7, followed by a decrease on day 14. Though the subsequent increase observed on day 21 in control group was not seen in other groups, the inhibition was significant only in the group receiving MS (*P*<0.05). The present study thus demonstrates that MS is effective in reducing the joint swelling in complete Freund’s adjuvant induced arthritis in rats.

**411**

**Analgesic activity of aqueous extract of *Plantago erosa* wall in albino mice**

Marak M, Akham S, Rajkumari B

**Regional Institute of Medical Sciences, Imphal, India.**

**Introduction:**

*Plantago erosa* Wall (PEW) belongs to family Plantaginaceae. It is a glabrous perennial herb, very common in the valley and hills of Manipur. It has been used by traditional practitioners in treating fever, boils, dysentery, diarrhoea and various inflammatory conditions.

**Methods:**

PEW was studied for analgesic activity by using acetic acid induced writhing in mice. Healthy albino mice weighing between 25 to 30gm were divided into five groups of six animals in each group. The animals were pre-screened before hand. PEW in doses 100, 200, 400mg/kg were given orally to different groups of mice. 2% gum acacia suspension in distilled water was used as control and aspirin 100mg/kg was used as standard. Writhing was induced by intraperitonial injection of 3% acetic acid in distilled water. Data was analysed for statistical significance using one way ANOVA followed by Dunnett’s ‘t’ test.

**Results:**

All the test doses of PEW (100, 200 and 400mg/kg) showed significant inhibition (*P*<0.01) of writhing movements. Standard drug aspirin showed the maximum inhibition.

**Conclusions:**

The present study shows that the aqueous extract of *Plantago erosa* Wall has significant analgesic activity.

**412**

**(-)-Nyasol from *Anemarrhena asphodeloides*is an inhibitor of eicosanoid and no production**

Lim H^1^, Nam JW^2^, Seo E-K^2^, Kim YS^3^, Kim HP^1^

**^1^Kangwon National University, Chunchon, Republic of Korea; ^2^Ewha Womans University, Seoul, Republic of Korea; ^3^Seoul National University, Seoul, Republic of Korea.**

**Objective:**

To assess the anti-inflammatory activity of constituents from the rhizomes of *Anemarrhena asphodeloides*, (-)-nyasol {*cis*-hinokiresinol, 4, 4’-[1Z, 3R]-3-ethenyl-1-propene-1, 3-diyl] bisphenol} was isolated and its anti-inflammatory activity was evaluated *in vitro* and *in vivo*.

**Materials and Methods:**

The anti-inflammatory activity of (-)-nyasol was examined in lipopolysaccharide (LPS)-treated RAW 264.7 cells and A23187- treated RBL-1 cells. *In vivo* activity was measured using carrageenan-induced paw edema assay.

**Results and conclusion:**

At > 1 *μ*M, (-)-nyasol significantly inhibited cyclooxygenase-2 (COX-2)-mediated PGE_2_ production and inducible nitric oxide synthase (iNOS)-mediated NO production in LPS-treated RAW 264.7 cells, a mouse macrophage-like cell line, but did not affect the expression levels of COX-2 and iNOS. (-)-Nyasol also inhibited 5-lipoxygenase (5-LOX)-mediated leukotriene production in A23187-treated RBL-1 cells. Furthermore, (-)-nyasol potently inhibited carrageenan induced paw edema in mice (28.6 - 77.1% inhibition at 24 - 120 mg/kg). Therefore, (-)-nyasol is a potential new lead compound and may contribute to the anti-inflammatory action of *A. asphodeloides*, possibly by inhibiting COX-2, iNOS and 5-LOX.

**413**

**Evaluation of anti-inflammatory activity of leaf extract of *Emblica officinalis***

Pandey SK^1^, Adiga S^2^

**^1^Sikkim Manipal Institute of Medical Sciences, Gangtok, ^2^Kasturba Medical College, Manipal, India.**

**Introduction:**

*Emblica officinalis* syn *Phyllanthus Emblica* (family: Euphorbiaceae) is among the most important plant in the Materia Medica. Different parts are used for various actions like Refrigerant, diuretics, astringent etc. Inflammation is a protective response intended to eliminate the initial cause of cell injury as well as the necrotic cells and tissues resulting from the original insult. It is not, a disease, but is usually a manifestation of disease.

**Methods:**

The leaves of *Emblica officinalis* were identified, collected and shade dried. The dried leaves were powdered and passed through mesh no. 40 to get moderately fine powder and extracted by petroleum ether followed by 50% aqueous methyl alcohol. The Anti-inflammatory activity was investigated using carrageenan induced rat paw edema and cotton wool pellet wool granuloma Model. It was tested in four group of rats (n=6) in each model using two dose 200mg/kg and 400mg/kg of test drug. Indomethacin 10mg/kg and 0.5%w/v sodium CMC 2ml were used as standard and control respectively.

**Results:**

The test compound showed 37.57% and 37.88% inhibition of inflammation in carrageenan model and 37.4% and 38.8% was seen in cotton wool pellet granuloma model at dose 200mg/kg and 400mg/kg respectively. Indomethacin 10mg/kg showed 42.38% and 43.6% inhibition of inflammation in carrageenan and cotton wool pellet granuloma model respectively.

**Conclusions:**

On the basis of above finding it may be concluded that aqueous methanolic extract of leaves of *Emblica officinalis* has anti-inflammatory activity. The results obtained were significant and are in agreement with the use in inflammatory conditions.

**414**

**Potent antiallergic/antiasthmatic activity of plant extract 4397**

Nath A, Maurya R, Raghubir R

**Central Drug Research Institute, Lucknow, India.**

**Objective:**

The effect of plant extract 4397 was evaluated and compared with (DSCG) for antiallergic / antiasthmatic in rats and guinea pigs.

**Methods:**

Passive cutaneous anaphylaxis (PCA) and mast cell stabilizing activity in SD rats and Schultz dale test in sensitized guinea pig were carried out to assess antiallergic / antiasthmatic activity of alcoholic extract of plant 4397.

**Results:**

Plant extract 4397 at the dose of 25, 50 and 100mg /kg PO showed 66%, 76% and 82% inhibition in PCA, which was very similar to 65%, 77% and 85% respectively at same doses of DSCG. Daily administration of plant extract at dose 5 and 10mg/kg PO for 5 days inhibited 50% and 77% respectively histamine induced mast cell degranulation. DSCG (10mg/kg ip for 5 days) inhibited 74% of mast cell degranulation in rats. The inhibitory effect on contraction of presensitized guinea pig ileum with egg albumin was taken a criterion for antiashthmatic activity. Plant extract at the dose of 0.5*μ*g/ml and 1.0*μ*g/ml showed 56% and 73% inhibition respectively. DSCG at the same doses caused 45% and 71% inhibition.

**Conclusions:**

It may be concluded that plant extract 4397 at same doses seems to have a very potent antiallergic / antiasthmatic activity in various test models, which is comparable to that of DSCG, a clinically used antiallergic drug. Further, studies are underway to characterize the single molecule from active extract for this bioactivity.

**415**

**To study the analgesic action of *Adhatoda vasica* on experimental animal models**

Mukherjee A, Das S

**Assam Medical College, Dibrugarh, India.**

**Introduction:**

Pain is an unpleasant sensation. It is actually a protective mechanism for the body. It occurs whenever any tissues are damaged, and it causes the individual to react to remove the pain stimulus.*Adhatoda vasica*, commonly known as vasaka or Malabar nut, is a medicinal plant belonging to the family Acanthaceae. It is generally used as an expectorant, oxytocic, anti-inflammatory and abortifacient.

**Methods:**

Ethanolic extract of the leaves of *Adhatoda vasica* (ELAV) was prepared by percolation method and subjected to oral toxicity testing using OECD guidelines. Central and peripheral analgesic actions were assessed with tail flick method and acetic acid induced writhing test respectively. 18 healthy animals (Albino rats for tail flick and albino mice for writhing test) were divided into three equal groups. Group1 received normal saline (10 ml/kg), group 2 received ELAV (500mg/kg) and group3 received standard drugs. Pethidine (5mg/kg) and aspirin(100mg/kg) were used as standard drugs for central and peripheral actions respectively. Statistical analysis was done using one way ANOVA followed by Dunnett’s multiple comparison tests. *P* value of < 0.05 was taken as significant.

**Results:**

ELAV showed significant increase in reaction time with tail flick method and significant decrease in writhing response when compared to control group. Concurrent treatment with the opioid antagonist, naloxone, partially reduced the antinociceptive effect of *Adhatoda vasica* indicating the involvement of endogenous opioid peptide in this action.

**Conclusions:**

The above study showed that ELAV possesses significant central and peripheral analgesic activity.

**416**

**Efficacy of dexamethasone in chronic phorbol esterinduced psoriasis-like lesions in mice**

Ravisankar R, Singh SK, Ramesh S, Sinha S, Nagpal J, Raj Kumar S, Ray A

**Ranbaxy Research Laboratories, Gurgaon, India.**

In this study we examined the effect of orally administered dexamethasone on TPA (12-O-tetradecanoyl-phorbol-13 acetate)- induced psoriasis-like lesions in mice, an animal model that shares histopathological, cellular, molecular and biochemical similarities with human psoriatic lesions. TPA (50nM) was topically applied on back skin of Balb/c mice three times per week for two weeks. Histologically, it elicited moderate to severe epidermal hyperplasia, hyperkeratosis and neutrophilic infiltration in the dermis. Further, increased expression of involucrin and transglutaminase 1 was demonstrated by immunohistochemistry. Additionally, four to forty fold increase in gene expression of MIP-2, MMP-9, cytokeratins K6 A, B and K16 and IL-6 was observed in TPA-treated animals as compared to control. Administration of dexamethasone-1mg/kg, once daily for the entire study period inhibited most of the TPAinduced pathological as well molecular changes in mouse skin. In conclusion, TPA-induced lesions in animals displayed several features of psoriasis and further support the clinical efficacy of steroids.

**417**

**Effect of polyherbal formulation (arthakure) in acute and chronic models of inflammation in rats**

Shah MB^1^, Patel AK^1^, Balaraman R^1^, Pandey AS^2^

**^1^Faculty of Tech. and Engg., The M. S. University of Baroda, Vadodara-390001. ^2^Herbal Body Cure Pvt. Ltd., C-278, Defense Colony, New Delhi, India.**

**Introduction:**

Present study was carried out to evaluate the antiinflammatory activity of Arthakure in both acute and chronic model of inflammation.

**Materials and Methods:**

Male Wistar albino rats (150-180g) were used in the study. Carrageenan -induced rat paw edema was used as acute model and Cotton pellets granuloma was used as chronic model for inflammation.

**Results:**

Treatment with Arthakure (500 and 1000mg/kg, p.o) showed a significant and dose dependent reduction in paw edema as compared to control group. The percentage inhibition of edema at 3 hrs was found to be 42.35% and 53.66% for doses 500 and 1000mg/kg, respectively. Treatment with Arthakure (500 and 750mg/kg, p.o.) showed a significant reduction in the granuloma formation surrounding the pellets. The percentage inhibition of granuloma formation was found to be 14.92% and 30.03% for doses 500 and 750mg/kg, respectively. In both the model of inflammation Arthakure showed significant anti-inflammatory action.

**Conclusions:**

Arthakure protects the acute and chronic phase of inflammation in dose dependant manner.

**418**

**Suppression of T helper responses by synergistic effects of PDE4 and PDE7 inhibitors: Implications for autoimmune disease therapy**

Vijayakrishnan L, Eapen MS, Balakrishnan B, Rudra S, Gupta N, Sattigeri J, Dastidar S, Bhatnagar PK, Ray A

**Ranbaxy Laboratories Limited, Gurgoan, India.**

A major impediment in the advancement of PDE4 inhibitors in clinical trials has been the dose limiting side effects of nausea, diahorrea and vomiting observed in this target class. On of the strategies aimed at achieving a safer drug profile has been to enhance the therapeutic window by targeting other PDE isoforms expressed in pro-inflammatory cells with the hope of maximizing efficacy. Previous studies have suggested that PDE7 inhibitors can attenuate anti-inflammatory activity mediated by PDE4 inhibitors. In order to delineate the mechanism of action of PDE4 and PDE7 inhibitors, we analyzed their effects on human CD4 T cells using *in vitro* immunopharmacological techniques. Our results show that, whereas PDE7 inhibitors when used alone fail to elicit inhibition of T helper cell responses, the synergistic effects of both PDE4 and PDE7 inhibitors significantly attenuated CD4+ T helper responses as reflected by their ability to inhibit TH1 cytokine IFNλ. This mechanism was found to be dependent on the ability of the inhibitors to modulate the function of antigen presenting cells. These novel findings suggest that designing hybrid drugs that co-inhibit PDE4 and PDE7 in inflammatory T cells can be utilized to achieve a drug profile with a higher therapeutic index. Dual PDE4/7 inhibitors may have a potential in the treatment of TH1 mediated autoimmune diseases like Multiple Sclerosis, Rheumatoid Arthritis, and Psoriasis etc.

**419**

**Antioxidant activity of alcoholic and ethyl acetate extracts of premna herbacea roxb. in Ehrlich’s ascitic carcinoma inoculated mice**

Ramalingayya GV, Pai KSR, Dhamija Isha, Setty MM, Manjula SN, Kumar Ravishankar

**Manipal College of Pharmaceutical Sciences, Manipal University, Manipal-576104, Karnataka, India.**

Oxidative stress has been reported to be one of the factors for tumor genesis and antioxidants have some significant retarding effect on cancer growth. In this context, the present study wanted to evaluate the possible antioxidant effect of the alcoholic extract of Premna herbaceae, a plant claimed to possess antitumor activity in indigenous medicine. *In vitro* antioxidant activity of title extract was tested in different models viz. DPPH radical scavenging assay, ABTS radical scavenging assay, o-phenanthroline assay, alkaline DMSO method, total antioxidant capacity and non-enzymatic hemoglobin glycosylation assay. *In vivo* antioxidant activity of alcoholic extract in EAC inoculate mice was carried by glutathione, thiobarbituric acid assay and catalase assay methods. The extract showed significant *in vitro* and *in vivo* antioxidant activity.

**420**

**Indian cinnamon - An immune system booster**

Niphade SR^1^, Mohd. Asad^2^, Gowda CK^3^

**^1^Bharati Vidyapeeth–s College of Pharmacy, CBD Belapur. Navi Mumbai. India. ^2^School of Pharmacy, Addis Ababa University, Addis Ababa, Ethiopia. ^3^Institute of Animal Health and Biologicals, Hebbal, Bangalore, India.**

The bark of Indian cinnamon (*Cinnamomum zeylanicum*) was studied for immuno modulatory effect using different experimental models such as neutrophil adhesion test, serum immunoglobulins test and indirect haemagglutination test. Cinnamon suspension was evaluated for both cell mediated and humoral immunity arms of immune system. The bark was administered as suspension by oral route at two doses 10 mg/kg and 100 mg/kg. Levamisole (2.5 mg/kg, *p.o*) was used as standard drug. The low dose of cinnamon bark (10 mg/kg, *p.o*) produced only an increase in serum immunoglobulins levels while the high dose of cinnamon bark (100 mg/kg, *p.o*) increased neutrophil adhesion, increased serum immunoglobulin levels and antibody titre values. Hence, this preliminary study supports the claim that cinnamon possesses immunostimulant activity.

**421**

**Ribonucleases as therapeutics**

Ahuja S, Pereira J.S.C, Kamat A

**Goa Medical College, Goa, India.**

Research on ribonucleases has already produced four Nobel prizes, signifies the ongoing research and their importance in cellular processes. Onconase, an RNase-based antitumor agent has reached phase IIIb clinical trials, and received orphan drug status from the U.S. FDA in January 2007 for treatment of mesothelioma.

Ribonucleases (RNases) are ubiquitously present in living organisms. They serve as enzymes for RNA degradation in almost all organisms. These include enzymes that hydrolyze single-stranded RNA or double stranded RNA, and RNA hybridized with DNA thus playing important roles in transcription as well as translation. However, these enzymes participate not only in RNA splicing & metabolism, but also in cell maturation, physiological cell death, angiogenesis, innate immunity against RNA viruses and have antitumor effects. Over the past two decades, scientific and technological innovations have led to up-rise in RNA-based therapeutics. Several classes of molecules and approaches have been investigated in RNA therapeutics as antisense RNA, ribozymes, ribonucleases, aptamers, small interfering RNA (siRNA) etc. RNases also form the central role in RNA interference therapy. This paper reviews various therapeutic potentials of natural and engineered with special focus as promising antitumor agents.

**422**

**RBx 33B4088C: A novel, potent muscarinic receptor antagonist for COPD**

Gupta Suman, Sinha Sandeep, Malhotra Shivani, Raj Kumar Shrimulla, Chugh Anita, Ray Abhijit, Sarma PKS, Kumar Naresh, Mohammad Salman, Kumar Bhatnagar Pradip Kumar

**Ranbaxy Research Laboratories, Gurgaon, Delhi, India.**

Chronic obstructive pulmonary disease (COPD) is a chronic and progressive disease of airway for which treatment options are very limited. Muscarinic receptor antagonists are the standard of care for symptomatic relief of COPD. RBx 33B4088C is a novel, potent muscarinic receptor antagonist with high affinity for m1 and m3 receptors as compared to the m2 receptors. When administered by intratracheal route, RBx 33B4088C has shown high *in vivo* potency and long duration of action for inhibiting muscarinic receptor agonist mediated bronchoconstriction in experimental animal models.

**423**

**Comparative clinical evaluation of diacerein and diclofenac in management of osteoarthritis of knee in Indian population- a prospective, randomized, open label, blinded end-point assessment trial**

Datta S, Tekur U, Kumar V

**Maulana Azad Medical College and Loknayak Hospital, New Delhi, India.**

**Introduction:**

Osteoarthritis (OA) is the most common joint disease in humans both in the western world as well as in India. Diacerein, a structural disease-modifying agent in OA (SYSDOA) has been marketed in India since 2006. We compared the efficacy, safety of diacerein with diclofenac in OA of knee patients.

**Methods:**

After one week wash-out period, subjects were randomized into two groups for 12 weeks treatment period with diacerein (50mg BD) or diclofenac (75mg BD) followed by 4 weeks of follow up. The primary outcome was a change in pain intensity after 20 meter walk measured by 100mm Visual Analog Scale (VAS). Secondary outcomes included change in WOMAC score, SF-36 quality of life questionnaire and Knee Society Score.

**Results:**

Twenty-five subjects in diacerein treatment group and 26 in diclofenac group were included in final analysis. VAS score in both groups significantly improved. At week 16, the score was significantly better in diacerein group. Diacerein treated subjects showed a significantly improved WOMAC score at week 8 and week 16. Consumption of rescue medication (Paracetamol) was significantly lower in diacerein group at week 12 and week 16. SF-36 scores however remained comparable in the two groups at the end of the study. One subject in diacerein group suffered from re-activation of pulmonary tuberculosis which was attributed to the study drug.

**Conclusions:**

Diacerein appears to have superior analgesic effect compared to diclofenac if given for prolonged periods. It also has a carry-over effect decreasing the need for analgesic medications once drug is withdrawn.

**424**

**Comparative study of the clinical efficacy of levamisole and betamethasone in progressive vitiligo**

Shyam Prasad A, Ramchandra S, Borah RN

**Kamineni Institute of Medical Sciences, Narketpally, India.**

**Background:**

Most probable pathogenesis of Vitiligo is Autoimmunity. Systemic corticosteroids suppress immunity and may arrest the progression of Vitiligo and lead to repigmentation. Levamisole seems to have immunomodulatory activity and restore depressed T cell functions. The present study was undertaken to compare the efficacy of Levamisole and Betamethasone in the management of Vitiligo.

**Materials and Methods:**

21 patients of Progressive Vitiligo with age ranging from 12- 65 years were taken in this study. A patient was considered improving if the lesions started re-pigmenting or the previously progressive nature has turned into stationary one.

**Regimens:**

Regimen I (10 patients) - Levamisole - 150 mg orally daily for 2 consecutive days each week, combined with topical Fluocinolone acetonide once daily. Regimen II (11 patients) - Betamethasone - 5 mg orally daily for 2 consecutive days each week, combined with topical Fluocinolone acetonide once daily. Patients were evaluated monthly for 6 months. Comparison of lesions was done using Digital Photography. Side effects were noted.

**Results:**

Arrest of progression in Regimen I was 80% and in Regimen II was 82%. Repigmentation in Regimen I was 70% and in Regimen II was 82%. Side effects with Regimen I were minimal. With Regimen II, weight gain, acne, mild gastric disturbance were noted.

**Conclusions:**

Here, the use of Levamisole is encouraging and if this regimen is used, then the side effects with steroid therapy can be averted. If Levamisole can restore the altered immune function then it may be more helpful in the treatment from the aetiopathogenetic point of view.

**425**

**Investigation of anti anti-asthmatic activity of ethanolic extract of *Thespesia populnea* bark (malvaceae)**

Patel Dipikaben^1^, Patel Vasantiben^2^, Patel NJ^3^, Patel N^3^

**^1^Radhe School of Pharmacy and Bio-research Institute, Vijapur, ^2^Krishna Institute of Pharmacy, Mehsana, ^3,4^S. K. Patel College of Pharmaceutical Education and Research, Mehsana, India.**

Bronchial asthma is a chronic inflammatory disorder of airways in which many cells and cellular elements play a role, in particular mast cells, eosinophils, T lymphocytes, macrophages, neutrophils and epithelial cells etc. *Thespesia populnea* is (Family: Malvaceae) a small or large tree; more commonly found in tropical and sub tropical area. *Thespesia populnea* is widely used traditionally in various inflammatory conditions as well as the anti inflammatory activity has been also proven scientifically. Anti-asthmatic effect of Ethanolic Extract of *Thespesia populnea* were carried out using histamine and acetylcholine induced bronchospasm in guinea pigs, Compound 48/80 induced mast cell degranulation and systemic anaphylaxis model in rats, Histamine induced contraction in guinea pig ileum, and Acetylcholine induced contraction in rat ileum. Histamine and Acetylcholine induced bronchospasm where preconvulsion dyspnoea was used as an end point following exposure histamine and acetylcholine aerosol. Significant Anti-Asthmatic activity was observed at 400 mg/kg dose of TPEE (Ethanolic Extract of *Thespesia populnea*) in histamine and acetylcholine induced bronchospasm in guinea pigs and systemic anaphylaxis in rats. Significant protection against mast cell degranulation was observed at 60 mg/ml dose of Ethanolic Extract of *Thespesia populnea* which was comparable with ketotifen. Spasmolytic activity was observed in Histamine induced contraction in guinea pig ileum; Acetylcholine induced contraction in rat ileum in dose dependant manner.

**426**

**Pioglitazone in experimentally- induced arthritis**

Mahajan P, Mediratta PK, Negi H, Banerjee BD

**University College of Medical Sciences and GTB Hospital, Delhi-110095, India.**

**Introduction:**

Rheumatoid arthritis, a chronic auto immune disorder, is characterized by accumulation of inflammatory cells in the synovium and destruction of the joint. Cytokines like TNFalpha and interleukin (IL)-1 promote the inflammatory response, while IL-4 inhibits the action of pro-inflammatory cytokines. Recently, peroxisome proliferator activated receptor (PPAR) has been shown to modulate and inhibit the inflammatory process. Thiazolidinediones are synthetic ligands that bind to PPAR-gamma and function by activating transcription of genes and also affect the inflammatory process. Pioglitazone, a thiazolidinedione was thereby chosen to study its effects on inflammatory process, cytokine production and arthritis in experimental models.

**Methods:**

Arthritis was induced by administration of Freund’s complete adjuvant (FCA) and motility and mobility were studied using the staircase climbing test. Inflammatory response was elicited by acetic-acid writhing test and carragenin- induced rat paw edema. Interleukin- 4 and TNF-alpha were estimated by the standard techniques.

**Results:**

There was a significant decrease (*P*<0.05) in the paw volume, tibiotarsal joint thickness, foot pad thickness and serum TNF- alpha levels by pioglitazone in a dose dependent manner but prednisolone produced more pronounced effects in comparable doses. However, IL-4 levels were raised equally by both pioglitazone and prednisolone.

**Conclusions:**

Pioglitazone appears to have significant anti-inflammatory and antiarthritic activity in the experimental models studied.

**427**

**Evaluation of activity of *Bombax malabaricum* and *Asparagus racemosus* in n-ethylmaleimide induced nflammatory bowel disease using Sprague-Dawley rats**

Trivedi ND^1^, Vidyasagar G^1^, Gandhi TR^2^, Patel KV^2^

**^1^Veerayatan institute of pharmacy, Mandavi, India; ^2^Ananad pharmacy college, Anand, India.**

**Objective:**

To evaluate the effect of *Bombax malabaricum* and *Asparagus racemosus* in N-ethylmaleimide induced inflammatory bowel disease animals.

**Methods:**

Male Sprague-Dawley rats (250gm) were allocated to 6 groups (n=6): Animals of Group I (normal control), Group-IV-VI were given water, Std drug (5-ASA 100 mg/kg), *Bombax malabaricum* (300 mg/kg) and *Asparagus racemosus* (200 mg/kg) respectively for 18 days once a day orally. On 11^th^ day of the study, inflammatory bowel disease was induced with 0.1 ml 3% N-ethylmaleimide (NEM) intracolonically in animals of Group-III-VI and Group II (vehicle control) received 0.1ml of 1% Methyl cellulose. During the study various physical parameter were calculated. On 18^th^ day, colon was removed, length and weight of it was measured and scored for histological parameters like Colon mucosal index (CMDI) and Disease activity index (DAI). Then homogenate was prepared for biochemical parameters Nitric oxide (NO), Malondialdehyde (MDA), Myeloperoxidase (MPO) and Superoxide dismutase (SOD). From each group one colon of randomly selected used for the histopathology study.

**Results:**

NEM model control animals showed significant reduction in body weight, daily water intake, food intake, colon length, SOD and increase Colon weight, MPO, MDA, NO, Microscopic, Macroscopic, CMDI, DAI and histopathological changes as compared to normal control. Pretreatment with 5-ASA, *Asparagus racemosus* and *Bombax malabaricum* reverse changes induced by NEM.

**Conclusions:**

Thus aqueous extract of *Bombax malabaricum* and *Asparagus racemosus* significantly reduce the severity of the IBD induced by 0.1ml 3% NEM intracolonically administration in rats. The protective activity might be attributed to anti inflammatory, anti oxidant and by healing properties of drugs.

**428**

**Evaluation of anti-inflammatory activity of ethanolic extract of *Acalypha indica Linn***

K Sandip, Manjunath PM, S Anitha, Goli Divakar, Sharma Uday Raj

**Acharya and B.M Reddy College of Pharmacy, Bangalore-560090, India.**

*Acalypha indica* was collected from the region of Hessaraghatta and was authenticated by the Department of Botany, Gnanabharathi, Bangalore. The fresh leaf of *Acalypha indica* was air dried and ground into fine powder. The finely powdered leaf was extracted with ethanol. The ethanolic extract was used for the evaluation of anti-inflammatory activity. 1% yeast suspension was used to induce inflammation in rat paw. The subcutaneous injection of 1% yeast induced inflammation in rats. The inflammation produced was measured plethysmometrically at the time intervals of 0, 1, 3, and 24 hours. The ethanolic extract was administered orally for the evaluation of anti-inflammatory activity. The significant (*P*<0.05) activity was observed at the dose of 200 mg/kg body weight after 24 hours. The percentage inhibition of paw oedema at 200 mg/kg body weight was found to be 71.42%.

**429**

**Hypoglycemic and anti-diabetic effect of ethanolic extract of *Euphorbia hirta* Linn. (Euphorbiaceae)**

Tamboli P, Patil P, Patil V, Surana S

**R.C.Patel Institute of Pharmaceutical Education and Research, Shirpur, India.**

The aim of this study is to assess the hypoglycemic and antidiabetic effect of Euphorbia hirta Linn. in alloxan-induced and fructose induced hyperglycemia in rats. Diabetes was induced by giving alloxan monohydrate (5 % w/v) intraperitoneally. Wistar rats weighing in between 180-240 gm were fasted overnight. They were randomly divided into the five groups and each group consists of six animals. Group A served as negative control. Groups B, C, D, and E were treated with ethanolic extract of plant *Euphorbia hirta* Linn. at the dose of 100, 200, 400 and 800 mg/kg of their body weight p.o. respectively for 21 and 30 days. It was found that ethanolic extract showed a significant decrease in blood glucose, serum cholesterol, triglyceride, creatinine level and increase in HDL level. The mean blood glucose concentration of control and extract-treated animals after oral administration of different doses of *Euphorbia hirta* Linn. at various time intervals (0, 30, 60, 90, and 120 min.) was found to be 79.31 and 78.42, 78.04, 76.48, 75.94 mg/dl respectively. Finally it was found that the extract of the plant *Euphorbia hirta* Linn. has the potential to act as anti-diabetic as well as hypoglycemic agent.

**430**

**Piperine acts as bioenhancer to potentiate the antiinflammatory activity of *Azadirachta indica* leaves (neem leaves) extract in rats**

Gavitre BB, Desai SK, Naik AB, Gawali VS, Ahmad A

**Principal K.M.Kundnani College of Pharmacy, Mumbai, India.**

**Introduction:**

Herbal medicine, an oldest form of healthcare known to mankind is an integral part of the development of modern civilization. *Azadirachta indica* (family: Meliaceae) commonly known as Neem tree is well known in India. Different parts of this medicinal plant have been reported to possess a wide range of pharmacological activities viz., anti-inflammatory, spermicidal, antiarthritic, antipyretic, hypoglycaemic, antifungal etc.

**Methods:**

Dried neem leaves were extracted with 80% ethanol using Soxhlet apparatus. Acute toxicity studies were carried out by administering the ethanolic neem leaves extract (NLE) p.o. to albino Wistar rats as per OECD-423 guidelines. Antiinflammatory activity of NLE was studied for acute (albumin induced paw edema) and chronic inflammation (cotton pellet granuloma). Rats were divided into six groups’ viz., control, standard drug and NLE (two dose levels) groups and the potentiation by piperine (two dose levels).

**Results:**

The NLE was found to be safe upto a dose of 5000 mg/kg, p.o. In albumin induced paw edema method a significant reduction in paw volume was observed in standard and NLE as compared to control group. NLE showed marked inhibition in granuloma formulation and lowered the elevated levels of serum lysosomal enzymes and lipid peroxidation as compared to control group which was further potentiated in the presence of piperine.

**Conclusions:**

NLE exhibited profound antiinflammatory activity in both (albumin induced paw edema and cotton pellet granuloma) animal models and its bioactivity is further enhanced in presence of piperine.

**431**

**Investigational role of hydrogen sulfide in adjuvant induced arthritis**

Patel SK, Modh VB, Patel DN, Patel JA

**Institute of Pharmacy; Nirma University of Science and Technology; Ahmedabad, India.**

**Introduction:**

Hydrogen Sulfide is recognized as an important mediator in neuromodulation, vasodilation and inflammation. The current study was planned to investigate the role of H_2_S in adjuvant induced arthritis in rats using sodium sulfide as H_2_S donor.

**Materials and Methods:**

Arthritis was induced in wistar rats by single subplantar injection (0.2 ml) of heat killed *Mycobacterium tuberculosis* (0.1 mg) in Freund complete adjuvant into left footpad. Intra peritoneal administration of Na_2_S (100*μ*mol/kg, 125 *μ*mol/kg, 150 *μ*mol/kg) and oral administration of standard drug dexamethasone 0.3 mg/kg were administered till 12 days and then rats kept untreated from 13 to 21 days. On day 21, severity of secondary lesions and parameters of inflammation were evaluated. Antioxidant parameters, tissue H_2_S levels and histology of hind limbs were performed after sacrificing the animals.

**Results:**

Following Na_2_S administration, rats showed dose dependent increase in inflammation compared to that in induced control and untreated sensitized animals. It produced marked infiltration of inflammatory cells compared to that of induced control group, cartilage destruction, vascular proliferation and pannus formation. Serum rheumatoid factor elevated in dose dependent manner when compared to induced control group. Na_2_S pretreatment increased Erythrocyte sedimentation rate, lowered Paw volume and showed endogenous antioxidant wasting compared to that of induced control group.

**Conclusion and Discussion:**

Our finding suggests that release of H_2_S aggravates adjuvant induced arthritis. The investigation on precise molecular mechanism involving the role of H_2_S in arthritis is being undertaken in our laboratory.

**432**

**Anti-inflammatory activity of leaf extracts of *Vitex altissima***

Anusha, Shareen Taj, Sudha Vani, Anitha, Ranganayakulu D

**Sri Padmavathi School of Pharmacy, Tiruchanoor, Tirupathi, India.**

**Objective:**

To evaluate acute and subacute anti-inflammatory property of different leaf extracts of *Vitex altissima* (Family: Verbenaceae) on male albino rats.

**Materials and Methods:**

Four different extracts of *Vitex altissima* namely hexane, chloroform, alcohol by soxhlet extraction and aqueous extract by percolation were prepared. These extracts were used to evaluate antiinflammatory property in acute model i.e., carageenan induced paw oedema model. Diclofenac sodium at a dose of 15 mg/kg body weight was used as standard. Further these extracts were also evaluated for their potential against subacute model of inflammation by cotton wool granuloma method. The obtained results were statistically validated using ANOVA followed by Dunnet’s test.

**Results:**

The oral administration of different extracts of *Vitex altissima* at a dose of 250 mg/kg body weight showed significant anti-inflammatory activity with aqueous (78%) and alcoholic extracts (44%). The inhibition being more prominent at the end of 2 hr was maintained till the end of 6 hr. The standard drug showed inhibition of 82% at the end of 2 hr. The local oedema caused by cotton wool implantation was effectively reduced by alcoholic extract of leaf of *Vitex altissima* (54%). But this reduction was more prominent over the standard drug Diclofenac sodium which caused only 34% inhibition. The other leaf extracts of *Vitex altissima* were unable to produce any pharmacological benefit in this subacute model.

**Conclusions:**

These results confirm the activity of leaf extracts of *Vitex altissima* against acute inflammation, thus supporting the experience of tribals who use this plant for ailments of shorter duration.

**433**

**Effects of polyherbal ayurvedic formulations on nonspecific immunity: An experimental study**

More D, Shetty V, Shah A, Mungekar S, Kamat SK, Rege NN

**Seth GS Medical College and KEM Hospital, Parel, Mumbai - 400012, India.**

**Introduction:**

To study effects of polyherbal Ayurvedic drug combinations [Tc and Pe (1:3) with or without Os coating] on non-specific immunity.

**Methods:**

Ethics committee permission was obtained. Effect of different concentrations of the test drugs was studied *in vitro* on chemotaxis and phagocytosis of human polymorphonuclear cells (PMN). Rats (n=100; either sex; 150-250 gm) were divided into 3 Gps, A, B, and C. Each group was further sub-divided into Gp1: Vehicle, Gp2:*Tc* (100 mg/kg/d), Gp3:*Tc+Pe* (400mg/kg/d) and Gp4:*Tc+Pe+Os* (400mg/kg/d). Study drugs were administered orally for 15 days. On day 16, in Gp A, alveolar macrophages were procured to assess NO release in presence and in absence of LPS. In Gp B, peritoneal macrophages were isolated to study their phagocytic activity. Gp C animals were subjected to cold immobilization stress (2 ½ hrs). Phagocytosis of peritoneal macrophages, adrenal weight and its ascorbic acid content was estimated.

**Results:**

Both combinations increased PMN phagocytosis, with maximum response at 600*μ*g/ml) (*P*<0.001). Though they significantly increased NO production, *Tc+Pe+Os* showed significantly higher NO production (*P*<0.001) than the other treatment groups. The combinations also increased peritoneal macrophage phagocytosis in normal as well as in animals exposed to stress. The increase in adrenal weight and decrease in ascorbic acid was prevented significantly (*P*<0.001) by both the combinations. Effect of combinations in stress was comparable to Tc alone.

**Conclusions:**

The polyherbal Ayurvedic drug combinations stimulated non-specific immune cells with activity higher than Tc alone. But when used against stress, their effects were comparable to Tc.

